# A Meta-Analysis on the Impact of High BMI in Patients Undergoing Transcatheter Aortic Valve Replacement

**DOI:** 10.3390/jcdd9110386

**Published:** 2022-11-09

**Authors:** Jiyoung Seo, Weijia Li, Israel Safiriyu, Amrin Kharawala, Sanjana Nagraj, Arooj Tahir, Ioannis Doundoulakis, Leonidas Koliastasis, Saul Rios, Leonidas Palaiodimos, Damianos G. Kokkinidis

**Affiliations:** 1 Department of Internal Medicine, Jacobi Medical Center/Albert Einstein College of Medicine, The Bronx, NY 10461, USA; 2 AdventHealth Orlando, Orlando, FL 32803, USA; 3 Department of Cardiology, Hippokration Hospital, National and Kapodistrian University of Athens, 10679 Athens, Greece; 4 Montefiore Einstein Center for Heart & Vascular Care, Albert Einstein College of Medicine, Montefiore Medical Center, The Bronx, NY 10467, USA; 5 Section of Cardiovascular Medicine, Yale New Haven Hospital, Yale University School of Medicine, New Haven, CT 06510, USA

**Keywords:** high body mass index, obesity paradox, transcatheter aortic valve replacement

## Abstract

Background: A paradoxical association of obesity with lower risk of transcatheter aortic valve replacement (TAVR) outcomes has been reported. We aimed to systematically review the literature and compare TAVR-related morbidity and mortality among individuals with overweight or obesity and their peers with normal body mass index (BMI). Methods: PubMed and Embase databases were systematically searched for studies reporting TAVR outcomes in different BMI groups. Separate meta-analyses were conducted for studies reporting hazard ratios (HR) and odds ratios/relative risks. Short- and mid-/long-term outcomes were examined. Results: 26 studies with a total of 74,163 patients were included in our study. Overweight was associated with lower risk of short-term mortality (HR: 0.77; 95% CI: 0.60–0.98) and mid-/long-term mortality (HR: 0.79; 95% CI: 0.70–0.89). Obesity was associated with lower risk for mid-/long-term mortality (HR: 0.79; 95% CI: 0.73–0.86), but no difference was observed in short-term mortality, although a trend was noted (HR: 0.87l 95% CI: 0.74–1.01). Individuals with obesity demonstrated an association with higher odds of major vascular complications (OR: 1.33; 95% CI: 1.05–1.68). Both overweight (OR: 1.16; 95% CI: 1.03–1.30) and obesity (OR: 1.26; 95% CI: 1.06–1.50) were associated with higher likelihood for receiving permanent pacemakers after TAVR. Conclusion: Individuals with overweight and obesity were associated with lower mortality risk compared to those with normal BMI but with higher likelihood of major vascular complications and permanent pacemaker implantation after TAVR.

## 1. Introduction

Obesity is a major public health concern with a disease burden that has tripled over the last 40 years [1]. In 2016, 650 million adults, which is 13% of the adult population globally, were estimated to suffer from obesity (BMI ≥ 30 kg/m^2^), and two billion people were estimated to be overweight (BMI 25–29.9 kg/m^2^) [1]. Obesity is a well-established risk factor for developing cardiovascular disease and a precursor to significant cardiovascular morbidity and mortality [2]. However, the impact of obesity in individuals undergoing cardiovascular interventions such as transcatheter aortic valve replacement (TAVR) is unclear. Despite the prevalence of obesity reaching nearly 15% in patients undergoing TAVR [3] and the annual TAVR volume in the United States overwhelmingly exceeding all forms of surgical aortic valve replacements (SAVR) (72,991 TAVR in 2019 vs. 57,626 surgical aortic valve replacement), the relationship between body mass index (BMI) and TAVR outcomes remains to be established [4].

Previously published reports evaluating the relationship between overweight or obesity and TAVR-associated mortality have reported conflicting results, wherein some studies paradoxically found individuals with obesity who underwent TAVR to have significantly better long-term survival rates compared to individuals with normal BMI and those who were underweight, an association commonly reported as the “obesity paradox” [5,6,7,8,9,10]. It has been hypothesized that this may be related to individuals with obesity being relatively younger and less frail and thus with a tendency to seek care early, be managed more intensively, and have lower comorbidity burden [5,6,7,11]. Conflicting data also exist in respect to short-term mortality and periprocedural complications. Some studies found an association between individuals with obesity and improved short-term survival after TAVR compared to their counterparts with normal BMI, while others found no significant difference [6,8,9,10,12]. Despite the lack of consensus in current literature, ascertaining the effect of overweight and obesity on TAVR outcomes is clinically relevant, as BMI can serve as a simple pre-procedural risk stratification tool during TAVR evaluation and potentially for other structural heart procedures. With this systematic review and meta-analysis, we aimed to evaluate the association of baseline overweight and obesity with periprocedural complications and with regards to short- and mid-/long-term mortality risk after TAVR.

## 2. Methods

This study was performed according to the Preferred Reporting Items for Systematic Review and Meta-Analyses (PRISMA) guidelines [13]. The study protocol was registered in PROSPERO (CRD42022350427).

### 2.1. Database Search and Study Eligibility

PubMed and EMBASE databases were systematically searched for eligible studies published up to June 26, 2022, by two independent researchers (JS and WL). The following search algorithm was adopted: (“transcatheter aortic valve implantation” OR “transcatheter aortic valve replacement” OR TAVI OR TAVR) AND (“body mass index” OR “body weight” OR BMI OR obes* OR overweight OR “body-mass index”). Reference lists of selected articles were additionally reviewed to identify eligible studies potentially excluded from our search algorithm. Any disagreements between two researchers were resolved by consensus of all the authors.

Studies were deemed eligible if (i) TAVR was performed in patients with aortic stenosis, (ii) the objective of the study was to report post-TAVR outcomes based on Valve Academic Research Consortium-2 definitions [14], (iii) comparative analysis based on their BMI was performed, and (iv) there were enough data reported to extract pertinent effect measures such as odds ratio (OR) or hazard ratio (HR).

Exclusion criteria were: (i) non-English language articles, (ii) duplicate patient population, (iii) lack of data on post-TAVR outcomes between different BMI groups, (iv) underweight population, and (v) surgical aortic valve replacement as the primary procedure performed. Case reports, editorials, reviews, conference abstracts and letters, guidelines, and study designs were excluded. There were no restrictions in terms of patient characteristics, sample size, or TAVR access approaches.

### 2.2. Data Extraction and Outcomes

Detailed information was extracted from each selected study by two independent reviewers (AK and AT) in a pre-defined data collection form. The following data were collected: (i) study characteristics (study design, location, study period, number of patients, BMI cutoff values, TAVI access approach, follow-up duration), (ii) patient baseline characteristics, (iii) primary outcomes, and (iv) secondary outcomes.

The primary outcomes were defined as short-term (30-day) mortality and mid-/long-term mortality (>1 year of follow up). The secondary outcomes included post-TAVI procedural complications defined as major bleeding, vascular complications, cerebrovascular events, myocardial infarction, new-onset atrial fibrillation, permanent pacemaker implantation, post-operative delirium, hospital readmission, and acute kidney injury.

### 2.3. Quality of Evidence Assessment

The Newcastle–Ottawa Scale (NOS) was used by two independent researchers (JS and AT) to methodologically assess the quality of non-randomized studies included in the analysis [15]. The NOS score ranges from 0 to 9 and three dimensions contribute to the overall quality score: selection of studies, comparability, and exposure [16]. A score of ≥7 denoted a high-quality study.

### 2.4. Statistical Analysis

Categorical variables were presented as frequencies or percentages, while continuous variables were listed in the form of means and standard deviations. The number of events, odds ratios (ORs), and hazard ratios (HRs) with corresponding 95% confidence intervals (CIs) were collected for primary and secondary outcomes. Bland’s method [17] was used to estimate the mean and standard deviation from the sample size, median, and interquartile ranges. Meta-analyses were carried out to compare groups among normal body weight, overweight, and obesity groups. Different BMI cutoff values were utilized when defining each body weight group in selected studies. Analyses were performed based on the group category defined in each article. Adjusted multi-variate ORs or HRs were prioritized for the analysis when available, and if not, unadjusted ORs with 95% confidence intervals were pooled using data from the original studies. Different meta-analyses were conducted for studies reporting HRs vs. ORs/event rates. We used the random-effects model (DerSimonian–Laird) for effect size estimation [18]. Statistical significance was defined as *p* < 0.05. Between-study heterogeneity was assessed through Q-statistic and Higgins I^2^ test, and high heterogeneity was indicated when *p* < 0.05 and I^2^ ≥ 50% [19]. Meta-regression analysis was performed to examine baseline variables with significant interaction with the outcomes. Funnel plots and Egger’s tests were used to assess publication bias, with p<0.05 indicating significant bias [20,21]. All statistical analyses were performed using STATA IC 17.0 (StataCorp LLC, College Station, TX, USA).

## 3. Results

### 3.1. Study Selection and Characteristics

**Figure 1:**
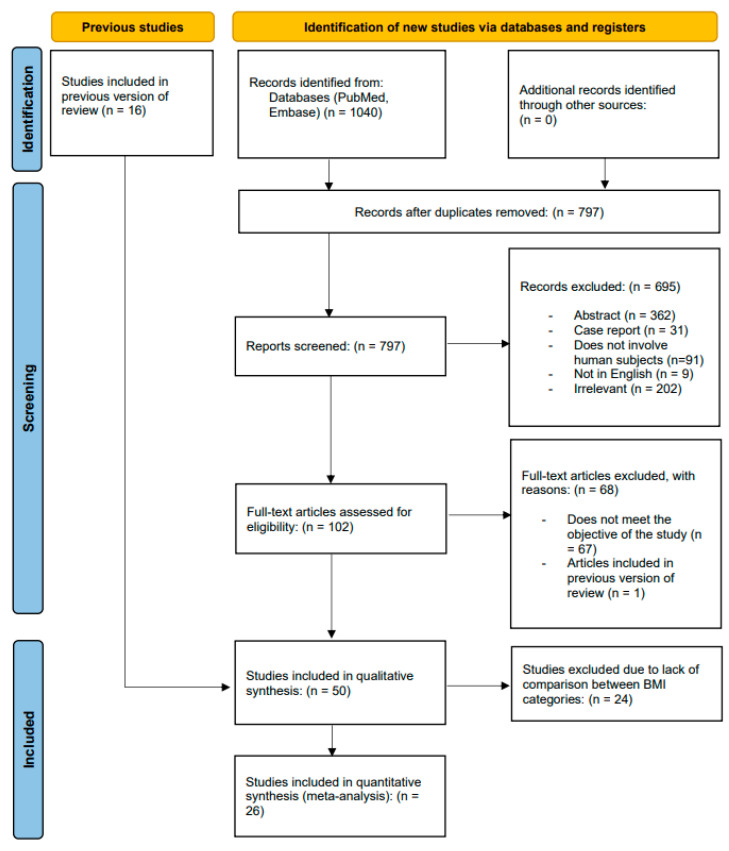
Preferred Reporting Items for Systematic Reviews and Meta-Analyses (PRISMA) flow diagram of the study selection process.

Of the 1040 records identified, 26 studies were eligible, with a total of 74,163 patients included in our analysis [7,9,10,12,22,23,24,25,26,27,28,29,30,31,32,33,34,35,36,37,38,39,40,41,42,43]. The PRISMA flow diagram is presented in Figure 1. All studies were retrospective observational studies published between 2013 and 2022, and follow-up duration ranged from 6 months to 5 years. The definitions of different weight groups were variable. Thirteen studies [7,9,22,23,24,29,32,36,37,39,41,42,43] categorized patients’ BMI (kg/m^2^) according to World Health Organization definitions [44]: underweight (BMI < 18.5 kg/m^2^), normal (BMI ≥ 18.5 kg/m^2^ and <25 kg/m^2^), overweight (BMI ≥ 25 kg/m^2^ and < 30 kg/m^2^), obesity (BMI ≥ 30 kg/m^2^). Overweight and obesity groups were younger with lower logistic European System for Cardiac Operative Risk Evaluation (EuroSCORE) and Society of Thoracic Surgeons (STS) score but had higher comorbidity (coronary artery disease, chronic obstructive pulmonary disease, diabetes mellitus, dyslipidemia, hypertension) rates compared to normal BMI group. Details of included studies and patient characteristics are summarized in Table 1 and Table 2, respectively.

### 3.2. Study Quality Assessment

The quality of all included studies was assessed using the Newcastle–Ottawa Scale (NOS), with a mean score of 7.9 (supplemental Table S1), suggesting that the included studies were of high quality.

### 3.3. Primary outcomes

#### 3.3.1. Short-Term Mortality

**Figure 2:**
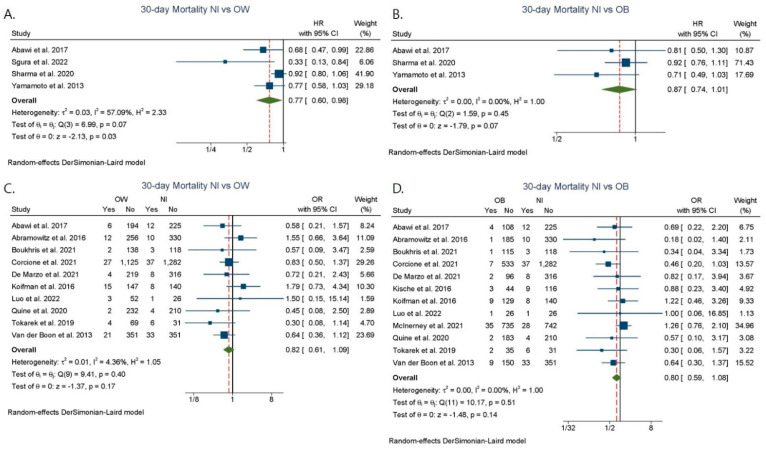
Short-term (30-day) mortality after TAVR among different BMI categories. (**A**) Normal BMI vs. Overweight. (**B**) Normal BMI vs. Obesity. (**C**), Normal BMI vs. Overweight. (**D**) Normal BMI vs. Obesity. HR: hazard ratio; Nl: normal BMI; OW: overweight; OB: obesity; OR: odds ratio [7,9,10,12,23,24,26,30,31,34,37,40,41,42,43].

HR as a measure of short-term mortality comparing patients with overweight vs. patients with normal BMI was available in four studies [24,40,41,43]. The analysis showed an association between overweight status and lower 30-day mortality risk (adjusted HR: 0.77; 95% CI: 0.60–0.98), but significant heterogeneity and publication bias existed (I^2^ = 57.1%, Egger test *p* = 0.0082) (Figure 2A). No difference was seen when we compared patients with obesity vs. patients with normal BMI [24,41,43] (adjusted HR: 0.87; 95% CI: 0.74–1.01, I^2^ = 0.0%) (Figure 2B). No significant differences were found between normal BMI versus overweight groups and obesity groups among studies reporting unadjusted ORs (Figure 2C,D).

#### 3.3.2. Mid-/Long-Term Mortality

**Figure 3:**
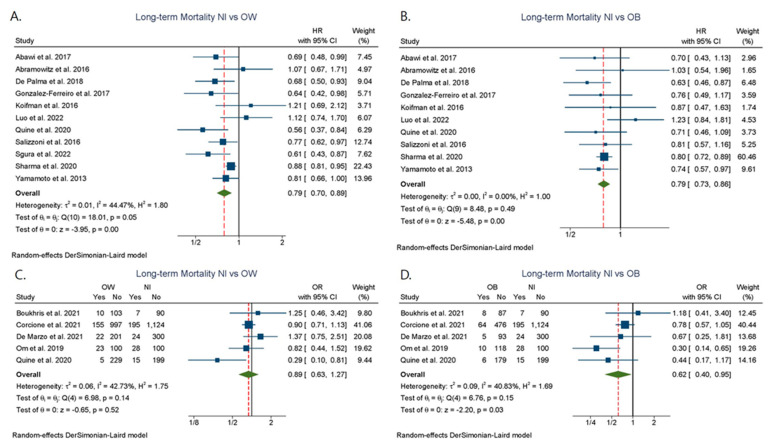
Long-term mortality after TAVR among different BMI categories. (**A**) Normal BMI vs. Overweight. (**B**) Normal BMI vs. Obesity. (**C**) Normal BMI vs. Overweight. (**D**) Normal BMI vs. Obesity. HR: hazard ratio; Nl: normal BMI; OW: overweight; OB: obesity; OR: odds ratio [9,12,23,24,26,27,29,31,34,35,37,39,40,41,43].

Eleven and ten studies compared the overweight and obesity groups, respectively, and provided adjusted HR for the comparison with normal BMI with regards to mid-/long-term mortality. Both the overweight (adjusted HR: 0.79; 95% CI: 0.70–0.89, I^2^ = 44.47%) and the obesity group (adjusted HR: 0.79; 95% CI: 0.73–0.86, I^2^ = 0.0%) had a significantly lower mid-/long-term mortality risk when compared to patients with normal BMI (Figure 3A,B). Pooled analysis of unadjusted ORs in five studies [12,23,26,35,37] confirmed this association for obesity (OR: 0.62; 95% CI: 0.40–0.95, I^2^ = 40.83%), but no difference was found with regards to the overweight group (OR: 0.89; 95% CI: 0.63–1.27, I^2^ = 42.73%) (Figure 3C,D). Only one-year mortality outcomes were pulled for unadjusted ORs, except for the Corcione [23] study, with a mean follow-up ranging 9.8 to 11.8 months.

### 3.4. Secondary Outcomes

**Figure 4:**
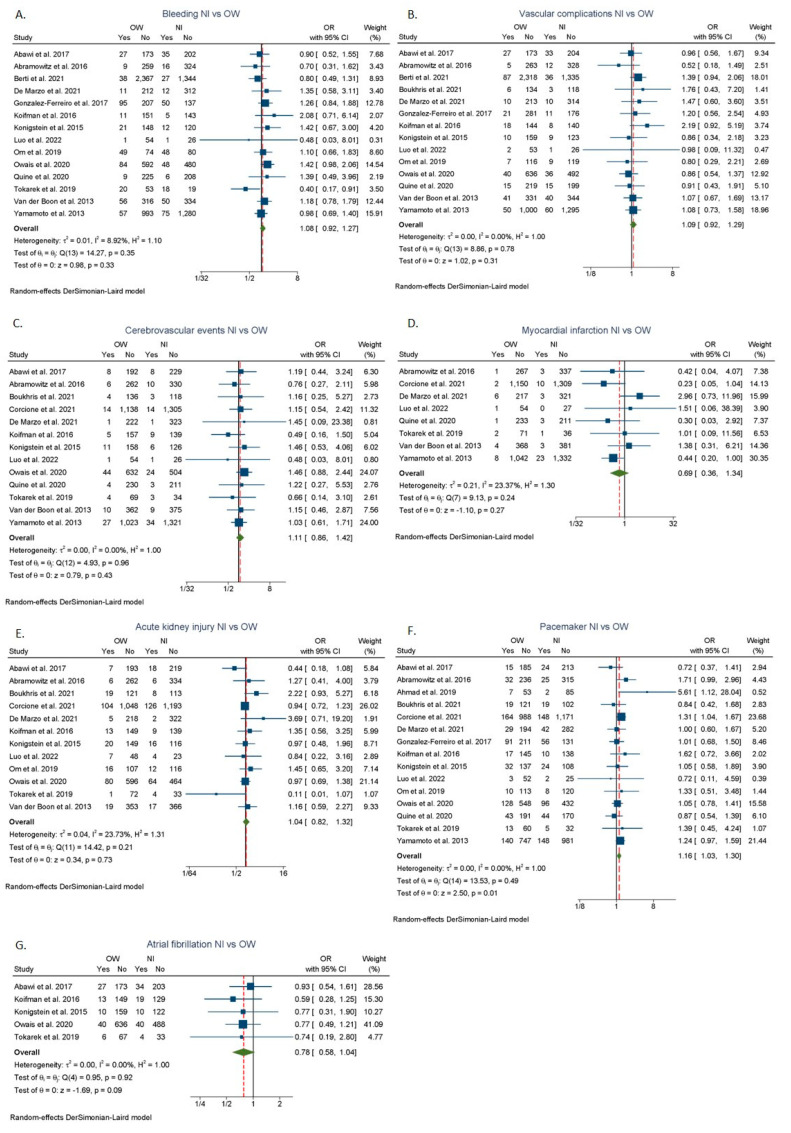
Complication rates after TAVR between normal BMI and overweight categories. (**A**) Major bleeding. (**B**) Major vascular complications. (**C**) Cerebrovascular events. (**D**) Myocardial infarction. (**E**) Acute kidney injury. (**F**) Permanent pacemaker insertion. (**G**) Atrial fibrillation. Nl: normal BMI; OW: overweight; OB: obesity; OR: odds ratio [7,9,12,22,23,24,25,26,29,31,32,34,35,36,37,42,43].

**Figure 5:**
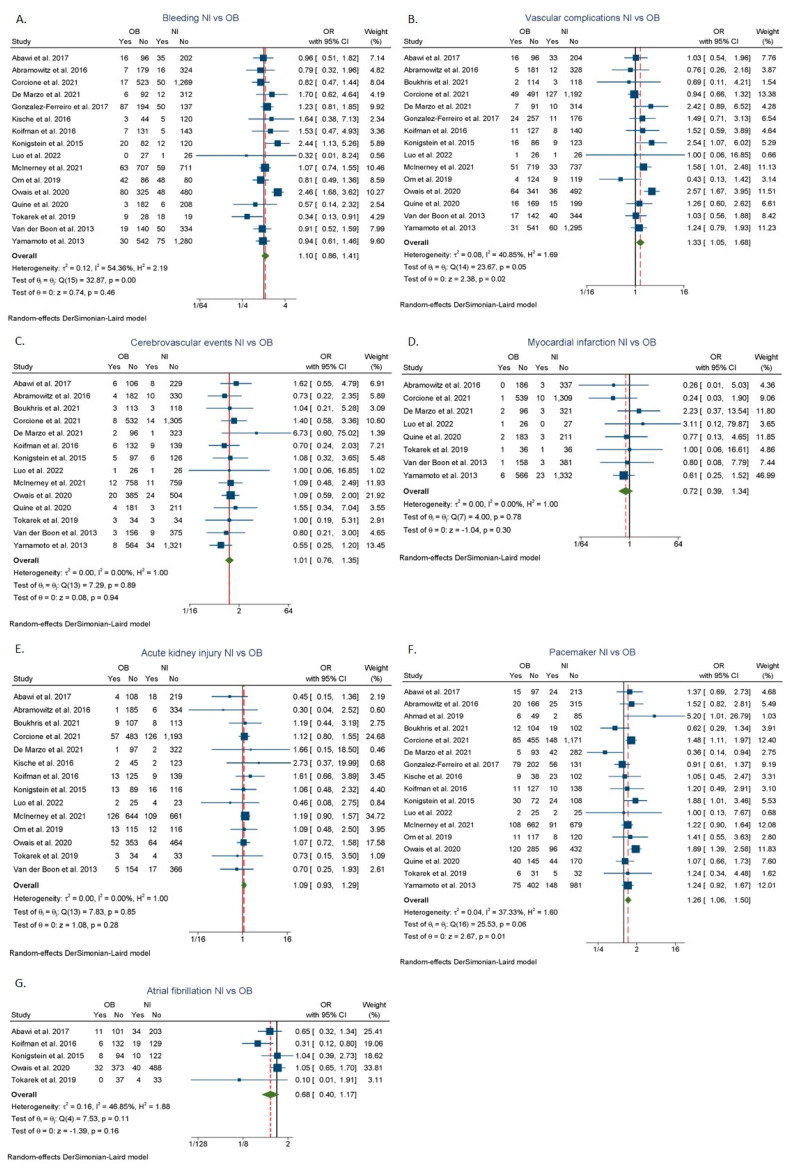
Complication rates after TAVR between normal BMI and obesity categories. (**A**) Major bleeding. (**B**) Major vascular complications. (**C**) Cerebrovascular events. (**D**) Myocardial infarction. (**E**) Acute kidney injury. (**F**) Permanent pacemaker insertion. (**G**) Atrial fibrillation. Nl: normal BMI; OW: overweight; OB: obesity; OR: odds ratio [9,10,12,23,24,25,26,29,30,31,32,34,35,36,37,42,43].

Patients with overweight had a higher likelihood of needing a permanent pacemaker implantation (OR: 1.16; 95% CI: 1.03–1.30, I^2^ = 0%) [7,9,12,23,24,25,26,29,31,32,34,35,36,37,43] (Figure 4F) as compared to patients with normal BMI. No significant differences were found in terms of major and life-threatening bleeding (Figure 4A), major vascular complications (Figure 4B), cerebrovascular events (Figure 4C), myocardial infarction (Figure 4D), atrial fibrillation (Figure 4G), and acute kidney injury (Figure 4E) in the overweight group. Patients with obesity were associated with significantly higher odds of major vascular complications (OR: 1.33; 95% CI: 1.05–1.68, I^2^ = 40.85%) [9,10,12,23,24,26,29,31,32,34,35,36,37,42,43] (Figure 5B) and need for permanent pacemaker insertion (OR: 1.26; 95% CI: 1.06–1.50, I^2^ = 37.33%) [7,9,10,12,23,24,25,26,29,30,31,32,34,35,36,37,43] (Figure 5F) compared to patients with normal BMI. No statistically significant differences were found in major and life-threatening bleeding (Figure 5A), cerebrovascular events (Figure 5C), myocardial infarction (Figure 5D), atrial fibrillation (Figure 5G), and acute kidney injury (Figure 5E) between obesity and normal BMI groups.

All primary and secondary outcomes are summarized in Table 3 and Table 4.

### 3.5. Publication Bias and Meta-Regression

The funnel plots and Egger’s test assessing for publication bias are presented in Supplementary Materials Figures S1 and S2 and Table S3, respectively. Only the comparison between overweight and normal BMI patients for short-term mortality showed significant publication bias (Egger test *p* = 0.0082). No significant associations between baseline characteristics and primary mortality outcomes were identified in our meta-regression analyses (Supplementary Materials Table S2).

## 4. Discussion

Our study presents a systematic review and meta-analysis on the effect of BMI on clinical outcomes after TAVR. The key findings of our study can be summarized as follows: (i) overweight status was associated with lower risk for short- and mid-/long-term mortality; (ii) obesity was associated with lower risk of mid-/long-term mortality; (iii) overweight and obesity were associated with higher risk of receiving permanent pacemakers after TAVR; and (iv) the obesity group was associated with a higher likelihood of major vascular complications.

While some studies have found one point increments in BMI (kg/m^2^) to be associated with progressively improved long-term mortality [5,6], other studies such as the one conducted by Gilard et al. [45] including 4571 TAVR patients found a higher risk of mortality with increasing BMI among patients with BMI > 32 kg/m^2^. Similarly, a study of 31,929 TAVR patients [41] showed that in patients with BMI > 30 kg/m^2^, a unit increase in BMI was associated with a 3% increased risk of short-term mortality. Both studies limited their analyses among patients with obesity. This probably shows that when studying the obesity-only sub-group, higher BMI is associated with worse outcomes. Contrary to that, when comparing any obesity or overweight status to patients with underweight or normal BMI, extra weight seems to be associated with protective effects. This can be explained by higher frailty or life-threatening diseases (i.e., end-stage cancer, advanced heart failure) associated with underweight and low-normal BMI populations both in procedures such as TAVR and under transcatheter interventions such as Transcatheter Edge-to-Edge Repair [11,46,47,48,49]. Although our study excluded the underweight population, there was inconsistency among included studies that compared frailty in different BMI groups: Abawi [24] and Berti [22] reported no difference between BMI groups; Tokarek [7] and Abramowitz [9] reported lower frailty in the obesity group compared to normal BMI and overweight counterparts; Luo [34] and Quine [37] reported rather higher frailty in the higher BMI group compared to other BMI groups. No included studies reported malnutrition data. Our study used multi-variate hazard ratios for the outcome of mortality to adjust for all these confounding factors when comparing normal BMI versus overweight and obesity groups. However, different studies adjusted for different characteristics and thus, even if the meta-analysis included adjusted HRs, this does not necessarily mean that it is adjusted for the same factors.

We found that obesity was associated with significantly higher likelihood of major vascular complications. It is interesting that our analysis shows that this is an issue only for the obesity group and not for individuals with overweight. This is expected, given how challenging femoral large-bore arterial access can be in individuals with significant fat tissue around groin. However, this was not shown by prior studies, with some of them finding no difference [5]. The Valve Academic Research Consortium (VARC)-3 strongly recommends recording detailed information regarding the access site and pre-planned vascular closure technique to better assess vascular complications in TAVR patients [50]. It also even provides different cut-off values for patients with obesity (BMI > 30) when assessing device success and prosthesis–patient mismatch, which is directly associated with all-cause and cardiac mortality [51]. This indicates that distinction should be made when studying outcomes of TAVR patients with different BMIs. Studies included in our meta-analyses, however, combined different access sites and vascular techniques, making it unclear whether specific access sites and/or vascular closure techniques are superior to others in limiting major vascular complication across different BMI categories.

An estimated 6–28% of patients undergoing TAVR receive permanent pacemakers [52]. The association between overweight or obesity and an increased risk of requiring permanent pacemakers after TAVR is not well-established in current literature. However, our study found both overweight and obesity to be associated with an increased risk of permanent pacemaker implantation after TAVR. Although multiple etiologies have been postulated to explain the occurrence of bradycardia requiring a pacemaker after TAVR (pre-existence of right bundle branch block, direct injury to atrioventricular and infranodal tissues, the use of self-expandable valve, male gender, baseline conduction abnormalities, larger prosthesis size, porcelain aorta, and increased implantation depth) [25,52,53,54,55], we suspect that larger prosthesis size requirements in patients with high BMI is probably contributing to our study findings [9,10,23,25].

Our study was unable to provide insights on the pathophysiology of obesity influencing favorable TAVR outcomes. Many possible mechanisms were introduced in other studies to explain the paradoxical phenomenon, including less frailty, younger population, early-on intensive medical interventions, protective peripheral body fat, and reduced inflammatory response with increased TNF-α receptors, but still the exact etiology remains unclear [5,6,9,56,57]. It is possible that the non-overweight/non-obesity groups had much higher likelihood of frailty, which is known to be associated with adverse outcomes post-TAVR [11,46], although no association between age and mortality was identified in our meta-regression analysis. It also remains unclear whether BMI can be adopted as the appropriate surrogate to investigate the true effect of overweight and obesity status of TAVR patients. Even if BMI is commonly used to define obesity, it is a relatively crude marker without accounting for the distribution of adipose tissue, especially visceral fat, which has been reported to have strong association with outcomes in cardiovascular disease, including for TAVR patients [58,59,60,61]. Future studies using other indicators such as body surface area (BSA) [62], pre-TAVR assessment of visceral abdominal fat using CT scan [10,63], waist-to-hip ratio of central obesity, or a combination of these may provide better understanding of obesity’s effect on TAVR outcomes.

## 5. Limitations

Our study has several important limitations. First, all studies included in our meta-analysis were retrospective in nature and were inherently susceptible to confounding factors. Second, the limited number of studies for certain important outcomes could have influenced the generalizability of our findings and precluded us from analyzing outcomes such as risk of readmission. Third, post-TAVR complications were derived from unadjusted, univariate data, which could not be adjusted by other confounding factors such as age or frailty. Frailty is known to be associated with negative outcomes after TAVR [64,65]. A multi-variate analysis would be helpful to draw additional conclusions. Fourth, various definitions of BMI categorization among all the included studies may have led to the heterogeneity observed in our analysis, necessitating the usage of standardized BMI classification (i.e., WHO definition) in future studies. Nonetheless, this study serves as the most updated meta-analysis on this topic to the best of our knowledge and highlights significant knowledge gaps and areas of future research.

## 6. Conclusions

Overweight and obesity—despite increasing the risk for vascular complications and permanent pacemaker insertion—were associated with improved survival likelihood after TAVR. Despite this strong association observed in the included observational analyses and in our systematic review and meta-analysis, we think that this might be driven by residual confounding by age, frailty, and other similar factors, which are more common in the normal or low BMI groups. We hope that future, well-designed, prospective cohort studies will shed light into this association and confirm whether there is a true obesity paradox or just unmeasured confounding.

## Figures and Tables

**Table 1:** Summary of the included studies.

Study	Year	Country	Study Characteristic	Study Population (n)	Follow-Up Duration (months)	BMI Classification (kg/m^2^)	Vascular Access
Abawi et al. [24]	2017	Netherlands	retrospective single center	562	12	WHO definition	Tf, Ta, Tao
Abramowitz et al. [9]	2016	USA	retrospective single center	805	33	WHO definition	Tf, Ta, Tao, S
Ahmad et al. [25]	2019	USA	retrospective single center	269	N/A	Underweight < 25 Normal 25 –≤ 30 Overweight 30 –≤ 35 Obesity ≥ 35	N/A
Berti et al. [22]	2021	Italy	retrospective single center	3776	N/A	WHO definition	N/A
Boukhris et al. [12]	2021	Canada	retrospective single center	412	12	Underweight < 20 Normal 20 –< 25 Overweight 25 –< 30 Obesity ≥ 30	Tf
Corcione et al. [23]	2021	Italy	retrospective single center	3075	(mean): 9.8–11.8	WHO definition	N/A
DeMarzo et al. [26]	2021	Italy	retrospective single center	645	12	Low to normal < 25 Overweight 25 –< 30 Obesity ≥ 30	Tf, Ta, S
DePalma et al. [27]	2018	Sweden	retrospective single center	493	36	Underweight < 18 Normal 18—25 Overweight 25.1–30 Obesity > 30 Severe obesity > 35	Tf, S, Ta, O
Gonska et al. [28]	2021	Germany	retrospective single center	611	N/A	BMI ≥ 25	Tf
Gonzalez-Ferreiro et al. [29]	2017	Spain	retrospective multi-center	770	36	WHO definition	Tf, Tax
Kische et al. [30]	2016	Germany	retrospective	172	12	Non-obesity BMI < 30 Obesity BMI ≥ 30	Tf
Koifman et al. [31]	2016	USA	retrospective single center	448	12	Low <20 Normal 20–24.9 Overweight 25–30 Obesity > 30	Tf
Konigstein et al. [32]	2015	Israel	retrospective single center	409	2	WHO definition	Tf
Lung et al. [33]	2014	France	retrospective multi-center	2552	1	Low < 18.5 Normal 18.5–29.9 Overweight BMI ≥ 30	Tf, Ta, S, O
Luo et al. [34]	2022	China	retrospective single center	109	35	Low < 21.9 Middle 21.9–27.0 High > 27.0	Tf, Ta
McInerney et al. [10]	2021	Europe, USA	retrospective multi-center	3174	24	Non-obesity 18.5–29.9 Morbidly obesity ≥40 or ≥35 with obesity-related comorbidities	Tf, non-Tf
Om et al. [35]	2019	Korea	retrospective multi-center	379	(median): 18.4 (IQR 7.3 to 37.2)	First tertile ≤ 22.3 Second tertile 22.4–24.8 Third tertile ≥ 24.9	Tf, Ta, Tao
Owais et al. [36]	2020	Germany	retrospective single center	1609	12	WHO definition	Tf
Quine et al. [37]	2020	Australia	retrospective multi-center	634	(median): 24	WHO definition	Tf, S, Ta, O
Saji et al. [38]	2022	Japan	retrospective multi-center	14472	12	Underweight < 20 Normal 20–25 Overweight 25–30 Obesity ≥ 30	Tf, non-Tf
Salizzoni et al. [39]	2016	Italy	retrospective multi-center	1904	(median): 25.7 (IQR 15.6 to 37.5)	WHO definition	Tf, Ta, Tao, Tax
Sgura et al. [40]	2022	Italy	retrospective multi-center	794	(median): 26.4	underweight < 20 Normal 20–24.9 Overweight/Obesity ≥ 25	Tf, Ta
Sharma et al. [41]	2020	USA	retrospective multi-center	31929	12	WHO definition	Tf, Ta, Tao, S, O
Tokarek et al. [7]	2019	Poland	retrospective single center	148	(median): 15.3 (IQR 6 to 34.7)	WHO definition	Tf, Ta, Tao, S
Van der Boon et al. [42]	2013	Europe	retrospective multi-center	940	(median): 12 (IQR 6 to 18)	WHO definition	Tf, Ta, S, O
Yamamoto et al. [43]	2013	France	retrospective multi-center	3072	(median): 4.1 (IQR 1 to 8.3)	WHO definition	Tf, Ta, S, O

BMI: body mass index; S: subclavian; Tao: transaortic; Ta: transapical; Tf: transfemoral; Tax: trans-axillary; O: others; IQR: interquartile range; WHO definition of BMI (kg/m^2^): underweight: (BMI < 18.5), normal: (BMI ≥ 18.5 and <25), overweight: (BMI ≥ 25 and <30), obesity: (BMI ≥ 30).

**Table 2:** Basic characteristics of normal, overweight, and obesity BMI patients.

	Normal	Overweight	Obesity
Age (years)	71.0 ± 24.3	65.9 ± 37.8	67.6 ± 19.1
Male n (%)	9198/19,032 (48.3)	10,187/18,919 (53.8)	4425/9571 (46.2)
BMI (kg/m^2^)	22.8 ± 2.6	27.6 ± 3.3	34.5 ± 5.9
AF n (%)	1191/3536 (33.7)	869/2947 (29.5)	776/2390 (32.5)
CAD n (%)	10,256/17,154 (59.8)	10,562/17,171 (61.5)	5433/8627 (63)
CKD n (%)	623/2847 (21.9)	898/3595 (25)	93/688 (13.5)
COPD n (%)	1306/6286 (20.8)	1140/5377 (21.2)	1021/3601 (28.4)
DM n (%)	2058/8277 (24.9)	2471/8228 (30)	1770/3989 (44.4)
Dyslipidemia n (%)	2780/5133 (54.2)	3283/5523 (59.4)	1668/2486 (67.1)
HTN n (%)	14,450/17,389 (83.1)	15,229/17,544 (86.8)	7994/8933 (89.5)
GFR (mL/min/m^2^)	57.1 ± 71.8	57.1 ± 71.0	53.1 ± 58.1
logistic EuroSCORE	18.0 ± 12.6	17.7 ± 11.9	14.5 ± 10.7
STS score	6.1 ± 3.7	5.3 ± 3.5	4.8 ± 2.6

Categorical variables are presented as frequencies and percentages, while continuous variables are listed in the form of means and standard deviations. BMI: body mass index; AF: atrial fibrillation; CAD: coronary artery disease; CKD: chronic kidney disease; COPD: chronic obstructive disease; DM: diabetes mellitus; HTN: hypertension; GFR: glomerular filtration rate; EuroSCORE: European System for Cardiac Operative Risk Evaluation; STS: Society of Thoracic Surgeons; SD: standard deviation.

**Table 3:** Summary of meta-analyses for all outcomes in normal BMI versus patients with overweight.

Outcomes		Studies	Patients	HR/OR	95% CI, *p*-Value	I^2^ (%)	Egger Test
Primary							
	30-day Mortality	4	25,050	0.77 (HR)	[0.60, 0.98], *p* = 0.03	57.09	0.0082
		10	6030	0.82 (OR)	[0.61, 1.09], *p* = 0.17	4.36	0.6904
	Mid-/long-term Mortality	11	28,917	0.79 (HR)	[0.70, 0.89], *p* = 0.00	44.47	0.8864
		5	3978	0.89 (OR)	[0.63, 1.27], *p* = 0.52	42.73	0.5304
Secondary							
	Major Bleeding	14	11,724	1.08 (OR)	[0.92, 1.27], *p* = 0.33	8.92	0.5321
	Major Vascular Complications	14	11,875	1.09 (OR)	[0.92, 1.29], *p* = 0.31	0	0.8544
	Cerebrovascular events	13	9940	1.11 (OR)	[0.86, 1.42], *p* = 0.43	0	0.3950
	Myocardial Infarction	8	7427	0.69 (OR)	[0.36, 1.34], *p* = 0.27	23.37	0.8140
	Atrial Fibrillation	5	2362	0.78 (OR)	[0.58, 1.04], *p* = 0.09	0	0.7519
	Pacemaker Insertion	15	10,071	1.16 (OR)	[1.03, 1.30], *p* = 0.01	0	0.9191
	Acute Kidney Injury	12	7338	1.04 (OR)	[0.82, 1.32], *p* = 0.73	23.73	0.9639

HR: Hazard ratio; OR: odds ratio; CI: confidence interval.

**Table 4:** Summary of meta-analyses for all outcomes in normal BMI versus patients with obesity.

Outcomes		Studies	Patients	HR/OR	95% CI, *p*-Value	I^2^ (%)	Egger Test
Primary							
	30-day Mortality	3	18,613	0.87 (HR)	[0.74, 1.01], *p* = 0.07	0	0.2995
		12	6461	0.8 (OR)	[0.59, 1.08], *p* = 0.14	0	0.0711
	Mid-/long-term Mortality	10	21,262	0.79 (HR)	[0.73, 0.86], *p* = 0.00	0	0.7745
		5	3173	0.62 (OR)	[0.40, 0.95], *p* = 0.03	40.83	0.8486
Secondary							
	Major Bleeding	16	10,042	1.1 (OR)	[0.86, 1.41], *p* = 0.46	54.36	0.3594
	Major Vascular Complications	15	10,033	1.33 (OR)	[1.05, 1.68], *p* = 0.02	40.85	0.4458
	Cerebrovascular events	14	9383	1.01 (OR)	[0.76, 1.35], *p* = 0.94	0	0.4143
	Myocardial Infarction	8	5804	0.72 (OR)	[0.39, 1.34], *p* = 0.3	0	0.7074
	Atrial Fibrillation	5	1876	0.68 (OR)	[0.40, 1.17], *p* = 0.16	46.85	0.0648
	Pacemaker Insertion	17	9878	1.26 (OR)	[1.06, 1.50], *p* = 0.01	37.33	0.5568
	Acute Kidney Injury	14	7485	1.09 (OR)	[0.93, 1.29], *p* = 0.28	0	0.2912

HR: Hazard ratio; OR: odds ratio; CI: confidence interval.

## Data Availability

Not applicable.

## Supplementary Materials

The following supporting information can be downloaded at: https://www.mdpi.com/article/10.3390/jcdd9110386/s1, Table S1: Newcastle–Ottawa scale; Table S2. Meta-regression analyses for primary outcomes in normal BMI versus patients with overweight and obesity; Figure S1. Funnel plots for mortality outcomes in normal BMI (Nl) versus patients with overweight (OW); Figure S2. Funnel plots for mortality outcomes in normal BMI (Nl) versus patients with obesity (OB).
