# Supplementary material for: A Meta-Analysis on the Impact of High BMI in Patients Undergoing Transcatheter Aortic Valve Replacement
Source: J Cardiovasc Dev Dis. 2022 Nov 9;9(11):386. doi: 10.3390/jcdd9110386 (PMC9695436; doi:10.3390/jcdd9110386)
Supplement: Supplementary file 1 [file jcdd-09-00386-s001.zip › jcdd-1953534-Table S1.pdf]

Supplemental Table S1. Newcastle-Ottawa scale

Study (Study, Year)	Representativeness of OW and OB	Selection of Nl	Ascertainment of Exposure	Demonstration of outcome of interest	Comparability	Assessment of outcome	Long enough follow-up	Adequacy of follow-up	Total Score
Abawi et al., 2017 [24]	1	1	1	1	2	1	1	0	8
Abramowitz et al., 2016 [9]	1	1	1	1	1	1	1	0	7
Ahmad et al., 2019 [25]	1	1	1	1	2	1	0	1	8
Berti et al., 2021 [22]	1	1	1	1	2	1	0	0	7
Boukhris et al., 2021 [12]	1	1	1	1	2	1	1	0	8
Corcione et al., 2021 [23]	1	1	1	1	2	1	1	1	9
De Marzo et al., 2021 [26]	1	1	1	1	2	1	1	0	8
De Palma et al., 2018 [27]	1	1	1	1	2	1	1	0	8
Gonska et al., 2021 [28]	1	1	1	1	2	1	0	1	8
Gonzalez-Ferreiro et al., 2017 [29]	1	1	1	1	2	1	1	0	8
Kische et al., 2016 [30]	1	1	1	1	1	1	1	0	7
Koifman et al., 2016 [31]	1	1	1	1	2	1	1	0	8
Konigstein et al., 2015 [32]	1	1	1	1	2	1	1	0	8
Lung et al., 2014 [33]	1	1	1	1	1	1	0	1	7
Luo et al., 2022 [34]	1	1	1	1	2	1	1	0	8
McInerney et al., 2021 [10]	1	1	1	1	2	1	1	0	8
Om et al., 2019 [35]	1	1	1	1	2	1	1	0	8
Owais et al., 2020 [36]	1	1	1	1	2	1	1	0	8
Quine et al., 2020 [37]	1	1	1	1	2	1	1	0	8
Saji et al., 2022 [38]	1	1	1	1	2	1	0	0	7
Salizzoni et al., 2016 [39]	1	1	1	1	2	1	1	1	9
Sgura et al., 2022 [40]	1	1	1	1	2	1	1	0	8

Sharma et al., 2020 [41]	1	1	1	1	1	1	1	0	8
Tokarek et al., 2019 [7]	1	1	1	1	2	1	1	0	8
Van der Boon et al., 2013 [42]	1	1	1	1	2	1	1	1	9
Yamamoto et al., 2013 [43]	1	1	1	1	2	1	1	0	8

Note: OW: overweight; OB: obesity; Nl: normal BMI group.
