# Supplementary material for: A Meta-Analysis on the Impact of High BMI in Patients Undergoing Transcatheter Aortic Valve Replacement
Source: J Cardiovasc Dev Dis. 2022 Nov 9;9(11):386. doi: 10.3390/jcdd9110386 (PMC9695436; doi:10.3390/jcdd9110386)

Supplemental Table S2. Meta-regression analyses for primary outcomes in normal BMI versus patients with overweight and obesity

	**Nl vs OW**				**Nl vs OB**			
	Thirty-day mortality		Long-term mortality		Thirty-day mortality		Long-term mortality	
	beta	p-value	beta	p-value	beta	p-value	beta	p-value
**Age**	0.0043	0.97	0.0255	0.88	-0.0392	0.78	-0.1569	0.54
**Male**	0.0004	0.80	0.0018	0.88	-0.0007	0.71	-0.111	0.54
**BMI**	0.1834	0.68	-	-	-0.436	0.47	-	-
**AF**	0.0038	0.39	0.0016	0.88	-0.0053	0.44	-0.0096	0.54
**CAD**	0.0026	0.22	-	-	-0.0026	0.43	-	-
**COPD**	-0.0004	0.83	0.0042	0.91	-0.0004	0.88	-0.0257	0.54
**DM**	0.00008	0.98	0.0153	0.89	-0.002	0.68	-0.0941	0.54
**Dyslipidemia**	-0.0001	0.99	-	-	-0.0051	0.62	-	-
**HTN**	0.0008	0.56	-	-	-0.0008	0.63	-	-
**GFR**	-0.0407	0.36	-0.0064	0.88	-0.0036	0.95	0.0392	0.65
**logistic EuroSCORE**	-0.0414	0.26	-	-	0.0088	0.87	-	-
**STS score**	0.4026	0.26	0.051	0.88	-0.148	0.81	-0.3138	0.54

Nl: normal BMI; OW: overweight; OB: obesity; BMI: body mass index; AF: atrial fibrillation; CAD: coronary artery disease; CKD: chronic kidney disease; COPD: chronic obstructive disease; DM: diabetes mellitus; HTN: hypertension; GFR: glomerular filtration rate; EuroSCORE: European System for Cardiac Operative Risk Evaluation; STS: Society of Thoracic Surgeons

Supplemental Figure S1. Funnel plots for mortality outcomes in normal BMI (Nl) versus patients with overweight (OW). Hazard ratios (HR) (A) and odd ratios (OR) (B) of thirty-day mortality; HR (C) and OR (D) of long-term mortality.

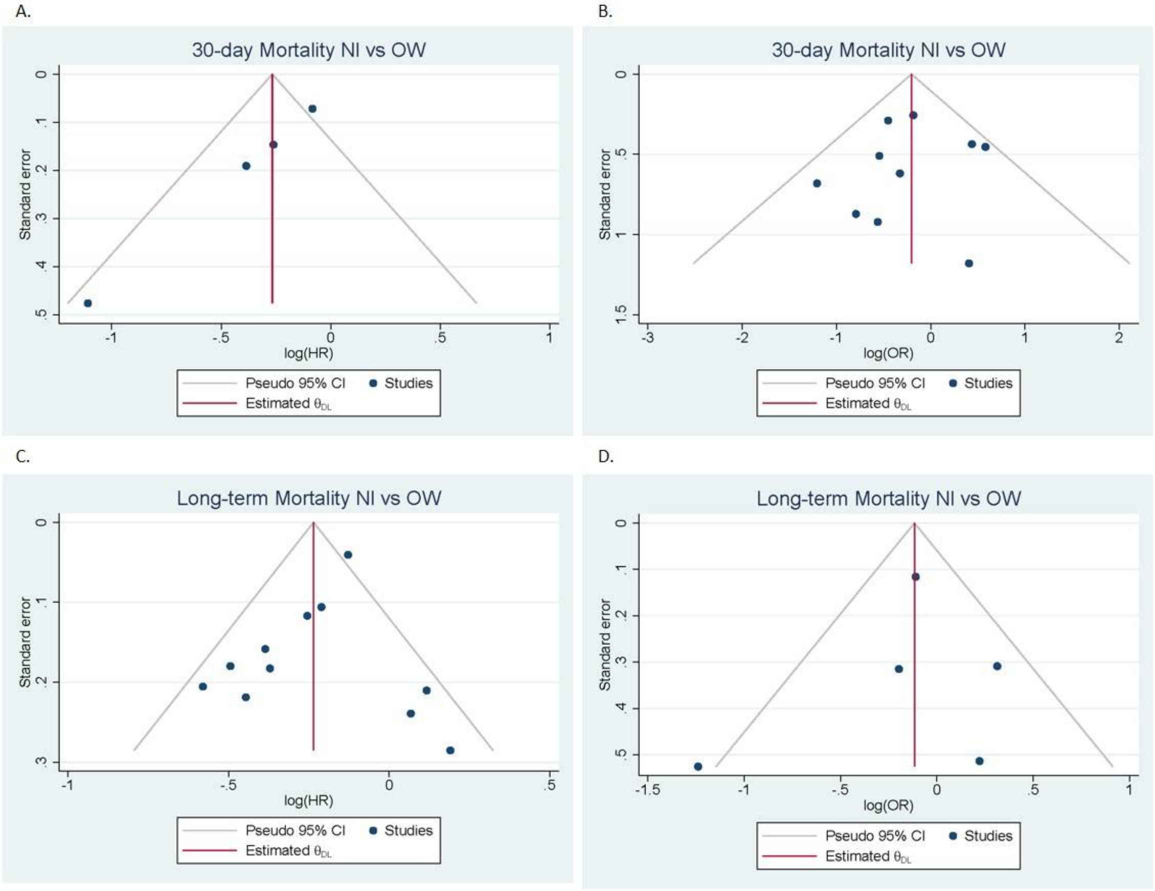

Supplemental Figure S2. Funnel plots for mortality outcomes in normal BMI (Nl) versus patients with obesity (OB). Hazard ratios (HR) (A) and odd ratios (OR) (B) of thirty-day mortality; HR (C) and OR (D) of long-term mortality.

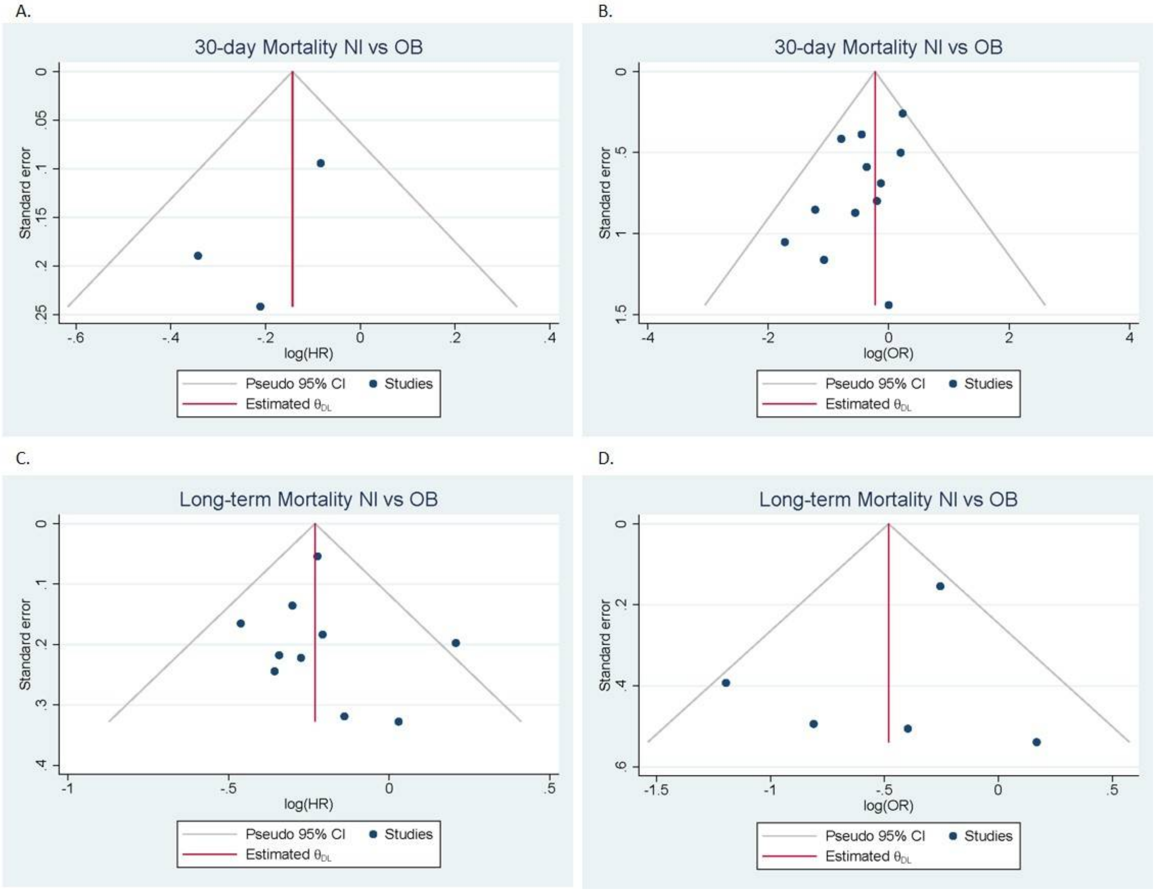

Supplement: Supplementary file 1 [file jcdd-09-00386-s001.zip › jcdd-1953534-Table S2 and figures.pdf]
